# Community-based approaches for malaria case management in remote communities in the Brazilian Amazon

**DOI:** 10.1590/0037-8682-0048-2020

**Published:** 2020-09-23

**Authors:** Jordi Gómez i Prat, Paulo Morais, Mercè Claret, Pere Badia, Romeo R. Fialho, Pedro Albajar-Vinas, Leopoldo Villegas, Carlos Ascaso

**Affiliations:** 1 Drassanes-Vall d’Hebron International Health Unit, International Health Programme of the Catalonian Institute of Health (PROSICS), Catalonia, Barcelona, Spain.; 2 Ministério da Saúde, Brasília, DF, Brasil.; 3 Project Manager Probitas Foundation, Barcelona, Spain.; 4 Site supervisor EE.RR. IDOM, Barcelona, Spain.; 5 Fundação de Vigilância em Saúde do Amazonas, Departamento de Vigilância Ambiental, Manaus, AM, Brasil.; 6 Department of Control of Neglected Tropical Diseases, World Health Organization, Geneva, Switzerland.; 7 Freelance, Washington, USA.; 8 Department of Public Health, University of Barcelona, Barcelona, Spain.

**Keywords:** Malaria, Diagnosis, Participatory action research, Community-based approach, Brazilian Amazon

## Abstract

**INTRODUCTION:**

Malaria case management is a pivotal intervention in malaria elimination. However, many remote areas in Brazil still lack access to basic health services. This study describes a community-based approach (CBA) for malaria case management in the large remote area of the Jaú National Park (JNP), Amazonas, Brazil.

**METHODS:**

In 2001, a general health CBA was initiated with a motor group (MG); a participative community health diagnosis (PCHD) was subsequently implemented between 2001 and 2005. In 2006, a CBA for malaria case management started with an expanded MG including all sectors with a stake in malaria control, from the local residents to the federal government. In 2008, community microscopists were selected and trained to diagnose hemoparasites. A full malaria strategy was implemented in 2009 with subsequent quality control follow-up.

**RESULTS:**

Two educational materials were co-created with local communities. The MG identified malaria as a major health problem and the malaria MG planned the control activities. Ten communities selected a resident to become malaria microscopists, and ten solar-operated health centers were built. The number of slide readings increased from 923 in 2006 to 1,900 in 2009, while malaria infections decreased from 354 cases in 2005 to 20 cases in 2015. The excess time (≥ 48 hours) between first symptoms and diagnosis/treatment decreased from 68.9% of cases in 2005 to 14.3% in 2010.

**CONCLUSIONS:**

While many factors were likely involved in the reduction of malaria transmission in the JNP, the CBA played an important role in the sustained success of the initiative.

## INTRODUCTION

It has been estimated that 138 million people were at risk of contracting malaria in the Americas in 2018. Malaria is endemic in 19 countries of the region with 929,000 cases and 580 deaths reported, along with a 14.0% increase in malaria cases from 2010 to 2018^1^.

Despite significant achievements, malaria remains a public health problem in Brazil, and the Amazon region is particularly affected. In 2018, 217,900 malaria cases and 44 malaria-related deaths were reported nationally^1^. In the last years up to 99% of the cases occurred in the Amazon region, where infections are typically concentrated around mining areas, timber extraction zones, and farming settlements, all of which cause environmental changes that favor malaria transmission^2^. 

Provision of health services in remote and sparsely populated areas of the Amazon basin remains a major challenge^3^, including increasing the availability of trained health workers, improving access to health facilities and the quality of their services, and logistical barriers. The Declaration of Alma Ata endorsed the involvement of community health workers (CHWs) for the co-creation in the planning, operation, and organization of health systems; community participation is another key element in strategies to overcome barriers in resource-limited settings^4^. The Brazilian Ministry of Health began a CHW program in 1991, officially instituted and regulated in 1997^5^; however, several operational challenges have arisen during its implementation, especially in remote areas of the Amazon. 

The Brazilian National Malaria Control Program (NMCP) has included multiple interventions, also the CHW, and operates through municipal-level teams as part of governmental decentralization. Applying malaria control measures in the Amazon basin is difficult given its specific characteristics, including its great geographic size, large dependence on river transportation, and low population densities. 

Ensuring access to prompt diagnosis of malaria and effective treatment remains a long-standing international goal and is fundamental to elimination-focused interventions^6^ ^-^ ^10^. This paper describes a community-based case management approach for malaria in remote areas of the Brazilian Amazon.

### Study area

**FIGURE 1:**
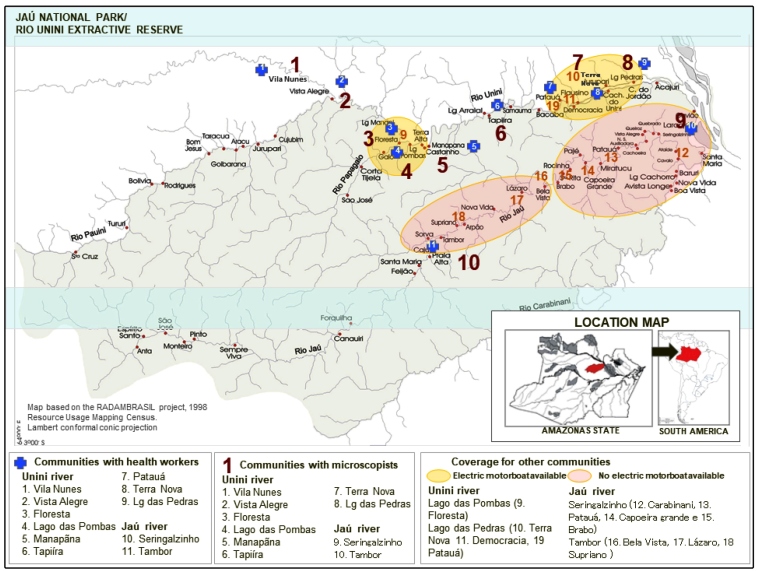
Location of the Jaú National Park in South America and distribution of community health workers, community microscopists, and health centers.

The Jaú National Park (JNP) is located in Amazonas on the bank of the Negro River, within the municipalities of Novo Airão and Barcelos, and 200 km north of the state capital, Manaus. The JNP measures 2,270,000 hectares; the Jaú River, a tributary of the Negro River, is the national park’s main river and divides it across the center (Figure 1). To the north are the Unini River and the Paunini River, a tributary of the Unini, while to the south lies the Carabinani River, a tributary of the Jaú. Communities in the JNP are largely established on the banks of the main rivers and their tributaries^11^. 

The population density of the JNP (0.04 inhabitants/km^2^) is one of the lowest in the country. The population decreased from approximately 920 inhabitants in 2001 to 788 in 2009; residents are distributed in 14 communities alongside the Unini (nine communities) and Jaú (five communities) rivers (Figure 1). The epidemiology of malaria in the JNP and the Barcelos municipality has been previously described^12^ ^-^ ^14^. A CHW program has been operating in the JNP since 2000.

## METHODS

The current project was based on Bordenave’s theory of learning^15^ ^,^ ^16^ and concepts of Participatory Action Research (PAR)^17^, with an emphasis on community engagement and the co-creation of the planning and creation of educational tools. The PAR was implemented in two cycles, with one focused on the general health and the other was specific to malaria case management . 

*First cycle: A community approach to general health:* In 2000, the inhabitants of the JNP claimed improvements in accessibility to the municipal health systems. In 2001, the first motor group (MG) was created and subsequently identified the need to conduct a health diagnosis and assessment. A participatory community health diagnosis (PCHD) project was conducted to identify major health needs of the JNP population. The PCHD used a quantitative and qualitative design, with both biomedical (e.g., nutritional status, vaccine coverage, intestinal parasitosis) and psychosocial components as topics in meetings with key informants, families, and communities. Between 2003 and 2004, the results were presented to and discussed with the local residents, and educational materials were co-created in two workshops where main health problems and action points were identified (Table 1). 

**TABLE 1:** Methodological components of the community engagement, results, and duration of activities of the first project cycle of the project at the Jaú National Park since 2001.

Key components of the Community Engagement	Description of methodological elements	Results observed	Years & duration of activities
1^st^ cycle: 2001-2005: Community Approach to General Health			
Human resource mobilization	**Creation of the Motor Group:** Configuration of a working group to coordinate all activities	Created a Motor Group with eight partners (one civil society, five governmental and two nongovernmental organizations).	2001 (nine months)
Health education	**Participative Community Health Diagnosis:** Quantitative and qualitative analysis of the health status and socioambiental determinants with health leaders and key informants from communities and families	Defined major health problems and socioambiental determinants	2001 (one-month field work)
Health education	**Community meetings:** Community workshops with communities and key community persons. *Discussion of results* in community meetings and with community health workers. *Preparation and drawing* up of manual of health community. *Community distribution and discussion* of printed general health material.	Co-creation of one educational tool (one manual of general health) done. Implemented strategy adapted to sociocultural and environmental realities. Raised awareness on prevention and community and personal self-care.	2003 (two-week field work), 2004 (one-week field work), 2005 (three-weeks field work).

*Second Cycle: Community approach to malaria case management:* After malaria was identified as the main health problem in the region, the MG was widened. In 2007, the MG members visited the JNP so that implementation details could be discussed with the communities and with the municipal health secretaries of Barcelos and Novo Airão. Between 2008 and 2009, community-level selection and the training of microscopists were implemented. Finally, between 2009 and 2010, quality control follow-up evaluations of the microscopists’ activities were conducted by the Amazonas state malaria control programme (SMCP) and a research team of the Tropical Medicine-Parasitology Laboratory of the Oswaldo Cruz Institute (Fiocruz). These agencies also conducted field worker trainings, including active searches for malaria cases (Table 2). 

**TABLE 2:** Methodological components of the community engagement, results, and duration of activities of the second project cycle of the project at the Jaú National Park since 2001.

Key components of the Community Engagement	Description of methodological elements	Results observed	Years & duration of activities
2^nd^ cycle: 2006-2010: Community Approach to Malaria Case Management			
Human resource mobilization	**Configuration Malaria Motor Group:** Configuration of a specific working group on malaria. Preliminary visit strategy.	Created a Malaria Motor Group with eleven partners (one organized civil society, eight governmental and two nongovernmental organizations).	2006 (a week-long planning meeting) 2007 (one-month field work)
Human resource mobilization & Health education.	**Selection and training of microscopists:** *Microscopists selection*. Process of selection based on pre-established criteria done by community members *Training of community microscopists* to diagnose hemoparasites, treat malaria and refer filariasis and Chagas disease cases, in coordination with the Malaria Control Programme of the state of Amazonas. *Preparation and drawing* up of manual of malaria.	Selected and trained ten inhabitants with new equipment provided. Co-creation of one educational tool (one manual of malaria)	2008 (one-month training)
Human resource mobilization, community structures building, equipment provision & health education	**Implementation of microscopists strategy in the field:** *Supply of Community structures, equipment, and consumables*. Building of local wooden health community centers, provision of microscopes and consumables needed for diagnosis, installation of solar power and batteries to ensure self-sustainable activities. *Community Meetings*. Meetings with communities and key community persons. Community distribution and discussion of printed malaria manual. *Start of microscopists activity*. Integration of malaria diagnosis and treatment procedures with the programmes at the state and national levels.	Ten new health centers with a room for microscopy reading built Community distribution and discussion of printed malaria material done.Activities of the ten microscopists started Implemented protocols, operational flows, monitoring mechanisms of diagnosis and antiparasitic treatment fully integrated with the malaria Control Programme of the state of Amazonas.	2009 (two-week field work), 2010 (two-week field work).
Human resource mobilization & Health education	**Follow-up of microscopists activity**: *Malaria quality control and refreshers courses for microscopists*: Quality control follow-up made by the malaria control programme for remote areas published by the Ministry of Health of Brazil. *In-service training and quality control*. In accordance with malaria control programme parameters and research activities conducted by the Oswaldo Cruz Foundation in the area, in-service training and quality control were carried out annually. Malaria active search by infected people and treatment. *Customized refresher courses* were provided wherever necessary.	Maintained level of good laboratory quality in malaria diagnosis.	2009 - 2010, every 4 months (total of 6 times), in-service training. 2009 (one-month refresher course)

*Data collection and indicators:* Main indicators for monitoring CBA malaria case management included the number of: 1. institutions involved; 2. community meetings and workshops; 3. co-created educational materials; 4. microscopists selected; 5. microscopists trained; 6. health centers built; 7. slides reading; 8. hours (time) between the start of symptoms and the diagnosis and treatment; 9. malaria posts reporting; and 10. the number of microscopy-confirmed malaria cases per 1,000 people per year. Malaria incidence data (2005-2015) were collected by the municipal health departments with detailed information organized by community, Plasmodium species, location, and year. All project data were analyzed using SPSS software, version 21.0 (IBM, Chicago, Illinois, USA).

### Ethical considerations

This study was approved by the ethics committee of the Vall d’Hebron University Hospital, Barcelona, Spain (Acta Number: 339, 18^th^ May 2018).

## RESULTS

### Community Approach to General Health

*Motor group configuration:* In 2001, an interdisciplinary MG was created with eight partnering organizations and institutions including: residents of the JNP; a Brazilian nongovernmental organization (NGO) specializing in socio-economic and environmental innovation in the Amazon (Fundação Vitória Amazônica); the health secretaries of Novo Airão and Barcelos; staff of the Alfredo da Matta Hospital representing the state health system; a Barcelos research campus of a federal research institution specialized in tropical medicine and public health (Fiocruz); an international NGO specializing in studies on the Amazon basin (Nucleus of Studies for the Amazonia of Catalonia-NeAC); and an international public health institution specializing in tropical medicine and international health (Unit of Tropical Medicine and International Health Drassanes, Catalan Institute of Health).

*Participative community health diagnosis: Needs assessment:* In 2001, the PCHD confirmed a high incidence of intestinal parasitic diseases (81%) along with low vaccination coverage (19.6%) in the JNP. Interviews with inhabitants revealed the most prevalent health problem to be malaria, followed by respiratory infections, diarrhea, dysentery, muscle and joint pain, hypertension, and skin diseases. Interviews with CHWs revealed that although blood samples (thick and thin smears) were regularly obtained from suspected malaria cases for diagnosis, prompt diagnostic results were usually unavailable. Hence, all suspected cases received a presumptive treatment for *vivax* malaria. If a fever persisted after three days, a new treatment was administered with the assumption of falciparum malaria. All slides were stained with Giemsa and sent to trained microscopists at the municipal health departments of Barcelos or Novo Airao; a long time-consuming process. Finally, interviews provided evidences that CHWs were in need of further training and technical and logistical support^18^ ^,^ ^19^. 

*Community meetings: Discussion of major problems and co-creation of educational materials:* Based on the PCHD and subsequent increases in community awareness and empowerment, two field public-health activities were implemented sequentially with the first being the co-creation (design, development, and distribution) of the health manual “Healthy Living in the JNP”^20^ (2003-2004). In 2005, feedback on the manual and discussions on the strategic action points led to agreement on a series of activities specifically adapted to local sociocultural and environmental conditions and context. The community meetings and workshops resulted in a raised awareness of community-level prevention activities and the need for personal self-care. All meeting and workshop participants agreed that malaria was the main problem to be addressed. 

### Community approach for malaria case management

*Malaria motor group configuration:* In 2006, with the objective of controlling malaria, the MG was broadened with the inclusion of representatives of the SMCP, the Fiocruz malaria research group in Barcelos, and the Tropical Medicine Foundation of Amazonas (FMT-AM). That same year, the MG held a one-week planning meeting in the city of Manaus to define the prevention and control malaria work plan. In 2007, field visits by the MG, community members, and key officials were held to create community-level inventories of available and needed buildings, equipment, supplies, and human resources. In addition, the groups selected their implementation strategies.

*Selection of community microscopists:* In 2008, ten people from ten target communities had been chosen for the microscopy positions and were officially recognized by the health municipal departments. Seven individuals were women and three were men. All were of legal age (21 to 39 years old), had received basic schooling, and could read and write. The selected microscopists were from the communities located on the Jaú River (Seringalzinho and Tambor) and on the Unini River (Lago das Pedras, Terra Nova, Manapana, Floresta, Lago das Pombas, Tapiíra, Vista Alegre, and Vila Nunes). Tapiíra was the only community that recruited a microscopist with prior training (Figure 1).

*Training of community microscopists:* The first microscopy training session was held between March and April, 2008. All participants travelled to the Barcelos city to receive training from NeAC and SMCP, in accordance with national malaria control standards. The course lasted 172 hours and took place over four weeks (Table 3). The course methodology considered the students’ prior and continued participation in their community’s co-creation and use of educational resources. Moreover, course’s contents, figures, and designs were used to develop a second manual called “Fighting malaria in the Jaú National Park and Rio Unini Extractive Reserve”^21^. Finally, all microscopists attended a 114-hour refresher course in January 2009 at the FMT-AM in Manaus. 

**TABLE 3:** Principal topics in the microscopy training sessions.

	Programme themes
1	Real stories relating to malaria
2	What we need to know about malaria
3	What we need to know when checking for malaria: the microscope, an important tool in the malaria examination
4	What we need to know to make the right malaria diagnosis: blood components, the plasmodium parasite life cycle, the different plasmodium species in Brazil
5	How to diagnose malaria using a microscope: thick and thin smears, collecting blood and preparing the slide, staining the slide, and counting parasites
6	Other parasites that might be found in human blood during an examination with an optical microscope (*microfilariae*, *Trypanosoma cruzi*)
7	Organizing the community health center
8	Personal safety in the laboratory

### Implementation of microscopy strategy in the field

*Supply of community structures, equipment and consumables:* In 2009, as part of the agreement between municipal health departments and the national SMCP, health center structures of two communities were improved and six new health centers were built. The new centers were built following the standards of the municipal health departments of Novo Airão and Barcelos. Eight centers (Lago das Pedras, Tapiíra, Manapana, Lago das Pombas, Vista Alegre, Vila Nunes, Seringalzinho and Tambor) were supplied with high quality microscopes, necessary supplies, solar power, and batteries to ensure that the activities could be performed in a sustainable manner. In 2010, the two remaining communities (Terra Nova and Floresta) also received new buildings, equipment, and supplies. Additionally, two electric motor boats were given to the communities of Lago das Pombas and Lago das Pedras to facilitate access to dwellings around the communities (Figure 1). 

*Start of microscopists’ activity:* Following the 2008 training, the microscopists participated in the building of their local health centers and began their work. In July 2009, a NeAC team visited all communities to install the solar panels and officially open the new health centers.

*Community Meetings:* During the NeAC visit in July 2009 the printed version of the second health manual, “Fighting malaria in the Jaú National Park and Rio Unini Extractive Reserve”^21^, was presented, distributed, and discussed with the community leaders and residents. 

### Follow-up of microscopists’ activity

*Quality control of malaria diagnosis:* Following the training, the microscopists were monitored according to the standard method used by the SMCP, with regular onsite tests and support from auditors. Diagnostic errors were observed at the beginning of the microscopists’ activities, but progressively decreased in number. The FMT-AM course conducted in January 2009 confirmed improvements and continued acceptable levels of diagnostic quality. 

*Microscopists’ in-service training and quality control:* The microscopists were evaluated quarterly during in-service trainings conducted in 2009 and 2010, and annually thereafter. The trainings were conducted by reviewers from Barcelos, Manaus or Rio de Janeiro, on the occasions of field research surveys developed by Fiocruz. After achieving optimal diagnostic quality, the microscopists increased the collaboration with the Fiocruz field research.

### Epidemiological data on malaria

**FIGURE 2:**
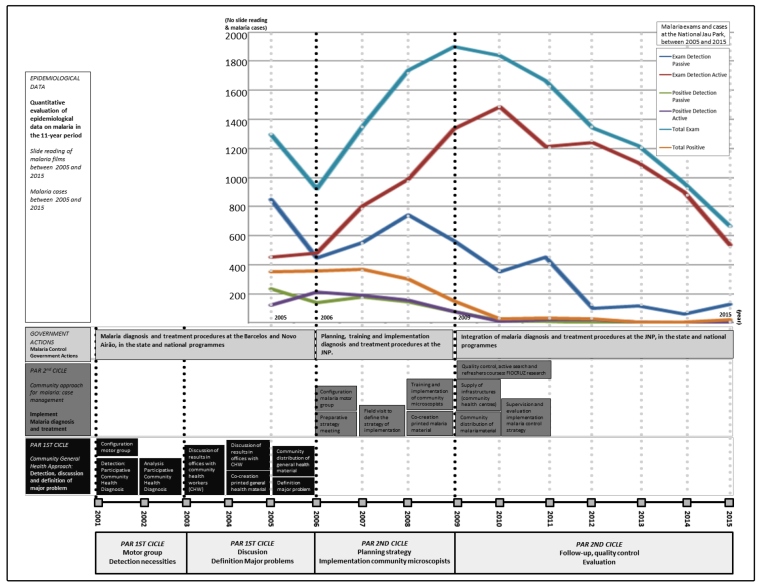
Timeline of project interventions at the Jaú National Park between 2001 and 2015, with epidemiological malaria data between 2005 and 2015.

*Slide reading of malaria films between 2005 and 2015:* A total of 1,299 slides were read in 2005 and 923 in 2006. By 2008, the number of slide readings reached 1,900, and in 2009 peaked above 1,900. In 2006, 51% of the slide readings had resulted from an active search for patients; this figure reached 70% in 2009 (Figure 2). Since 2009, the majority of the slide readings have been related to the Fiocruz research. 

*Prevalence of malaria cases between 2005 and 2015:* In 2005, 361 malaria cases were diagnosed (*P. vivax*: 59%; *P. falciparum*: 38%). In 2007, 455 cases were diagnosed (*P. vivax*: 61%; *P. falciparum*: 36%), falling to 187 cases in 2009 (*P. vivax*: 81%; *P. falciparum*: 18%), 33 cases in 2010 (*P. vivax*: 97%; *P. falciparum*: 3%), 3 cases in 2014 (*P.vivax*: 100%), and 20 cases in 2015 (*P. vivax*: 100%). The annual parasite index (API) fell from 469.54/1,000 residents in 2007 to 25.38 in 2015 (Figure 2). 

*Characteristics of malaria cases across the communities:* In general, all communities followed a similar pattern of decreasing infections. The most significant reduction in the number of malaria cases was observed in the community of Vista Alegre, with 118 malaria cases in 2009, 18 cases in 2010, and one case in 2015. In 2011, the highest numbers of cases were diagnosed in Lago das Pombas (four cases) and Patauá (seven cases). The community of Tambor had 22 cases in 2011, followed by 19 cases in 2012 and no cases in 2014. The malaria species and the origin (autochthonous or imported) of cases were recorded for each diagnosis. In 2009, we observed an increased number of 14 cases in Tapiíra, of which four were due to *P. falciparum*, one was due to a mixed species, and four were of imported origin.

*Time between the onset of symptoms and malaria diagnosis and treatment:* In 2005, before implementation of the intervention, the average percentage of diagnoses made within 24 hours of symptom onset was 8.6%; the average percentage of diagnoses made after 48 hours was 68.9%. In contrast, after implementation of the intervention, in 2008 these figures were 34.4% and 33.3%, respectively. In 2010, 73.9% of diagnoses and treatments were made in the first 24 hours after the onset of symptoms and 14.3% were made after 48 hours.

## DISCUSSION

Following Bordenave's theory of learning, based on the problematization of issues and participatory problem solving,^15^ ^,^ ^16^ the sharing and discussing of experiences and realities directly contributed to the eventual shared understanding and ownership of key community-level issues. Together with the use of PAR, participants were able to develop plans of action to improve local conditions that enabled malarial transmission. The community actions taken between 2001 and 2005 definitively stimulated and valued community engagement, involving residents in the diagnosis and the planning and implementation of health improvement strategies. The first MG relied on the participation of local residents, together with contributions from eight civic and governmental institutions. In 2005, the JNP population finalized a parallel empowerment process creating a civil society organization registered as the Unini River Residents’ Association (AMORU). Once the malaria work began in 2006, the number of MG institutions rose to four. This ensured the involvement of all relevant persons acting directly or indirectly in the JNP, facilitating project sustainability. From the initiative’s beginnings in 2008, the community health agents and microscopists actively participated in the various meetings and workshops, building their knowledge, their credibility with the population, and in turn increasing their self-esteem. The co-creation processes of the two manuals, along with joint planning of their use, ensured the accuracy of their contents and had an important impact on their wide-spread use. 

**FIGURE 3:**
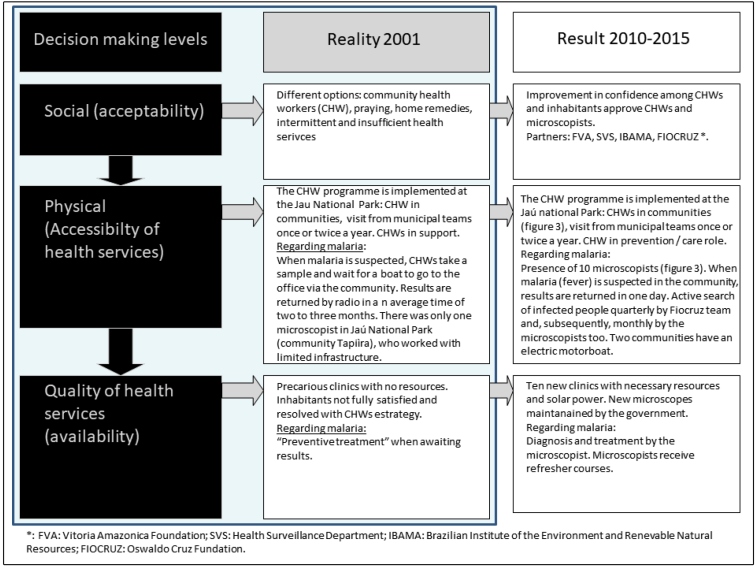
Discussion components at the three decision-making levels relating to the Jaú National Park comparison of 2001 and 2010.

Specifically considering malaria, informative, educational and strategic activities were conducted at the three decision-making levels of the health sector: 1. social - acceptability and decision to seek out healthcare; 2. physical - accessibility to a health service; and 3. service quality - availability, provision of adequate care, and the active searching for infections and diseases. These three levels are essential for improving accessibility to diagnosis and treatment^22^ ^,^ ^23^. The initial PCHD had uncovered failures in all three (Figure 3).

To improve activities at the social level, the communities participated actively in selecting the target health problem, planning the project, and recruiting and selecting the microscopists. Together with attending the educational lectures conducted during the community meetings and during the Fiocruz research surveys, the abovementioned factors were key to successfully reinforcing the importance of seeking health care at the onset of malarial symptoms among community members. The implementation of on-site diagnostic confirmation, including for other hemoparasites (i.e., filariasis and *Trypanosoma cruzi*), was an important added value for the population and for achieving malaria control^24^.

Improvements in malaria diagnoses at the physical level were marked in 2008 by inclusion of the community microscopists in the official teams of both municipalities. Further improvements occurred in 2009 with completion of fully equipped health centers. In addition, a major barrier in maintaining health centers in remote communities continues to be the considerable expenses for gasoline or diesel to generate the electricity necessary to operate the microscopes. Thus, providing solar energy with battery backup greatly enhanced self-sustainability and prevented drawing on the limited resources of the communities. It is also important to underline that the communities and microscopists easily adapted to the contextual and technological changes progressively introduced into the JNP after the study period, including broader access to electric power and rapid diagnostic tests. This showed that the main key factor for the sustainability of the microscopy activity was, rather than simple access to specific instruments, the collective and individual human investment (community participation, community microscopists).

Finally, regarding the service quality level, maintaining the ability to provide a diagnosis within 48 hours in remote areas is an important indicator of service quality. However, having a microscopist per community could be not attainable. The initial training course and the subsequent monitoring and refresher courses ensured the highest possible standards for microscopists. Periodic professional review of the slides (100% of positive and 10% of negative slides) was also essential for monitoring quality. A study developed by Salcedo^6^ between 1994 and 1996 reported how introducing microscopy to a community led to a reduction in the time between the onset of malarial symptoms and diagnosis and specific treatment from 3.5 to 1.3 days. In our case, in 2010, less than two years after starting community microscopy, we observed 73.9% of diagnoses made in under 24 hours and 14.3% within 48 hours. In this context, the importance of microscopists involveds: i) not unnecessarily treating reducing the treatment of false-negative cases; ii) not delaying the expediting treatment of *P.falciparum* infections; iii) ensuring follow-up of treated individuals; and iv) enabling the diagnosis of two other haemoparasites.

*The local epidemiology of malaria:* We observed a 94.6% reduction in the API, specifically, from 469/1,000 residents in 2007 to 32.9 in 2010 and to 25.38 in 2015. In the Salcedo study^6^, a reduction of 74.1% in the API was observed after microscopy was implemented. In the JNP, malaria transmission dropped dramatically after the introduction of the microscopists and the active searches for infected people conducted by Fiocruz. The overall number of malaria cases decreased from 354 in 2005 to 20 in 2015 - a 94.4% reduction. In contrast, for the whole state of Amazonas the reduction was 69.9% between 2007 and 2011^25^. Specifically regarding *P. falciparum* malaria*,* there was a very significant reduction from 139 cases in 2005 to no cases in 2015. 

This study has made the relevant challenges in a project of this kind evident. Hiring a large number of microscopists to focus on a limited number of endemic diseases was a significant challenge for municipal health services with limited financial resources. Without thorough planning, an otherwise appropriate strategy could have diverted assistance from other rural and urban areas, jeopardizing sustainability and preventing regional scaling-up. Introducing microscopists at strategic malaria hotspots throughout the municipality is an approach requiring thorough advance planning. 

Community engagement and education are crucial factors in the adoption and sustainability of strategies related to the malaria control, as observed in the study conducted in Mato Grosso^26^ There, an initiative was successful in encouraging people to visit a clinic immediately at the onset of fever. These results were corroborated by the experiences of a participatory educational programme in 2008 with teachers in the municipality of Barcelos^27^. International experiences have also shown community engagement and community microscopists to be key factors in malaria control^28^ ^,^ ^29^.

Finally, although other factors play a role in the long-term reduction of malaria cases, the introduction of community microscopists through community-based methodologies is an approach that should be considered by health authorities. In our case, the methodology was key for the successful establishment of malaria control in remote areas of the Brazilian Amazon.
